# Indispensable roles of OX40L-derived signal and epistatic genetic effect in immune-mediated pathogenesis of spontaneous pulmonary hypertension

**DOI:** 10.1186/1471-2172-12-67

**Published:** 2011-12-15

**Authors:** Moloud Rabieyousefi, Pejman Soroosh, Kimio Satoh, Fumiko Date, Naoto Ishii, Masahiro Yamashita, Masahiko Oka, Ivan F McMurtry, Hiroaki Shimokawa, Masato Nose, Kazuo Sugamura, Masao Ono

**Affiliations:** 1 Department of Pathology, Tohoku University Graduate School of Medicine, 2-1 Seiryo, Aoba-ku, Sendai, Miyagi 980-8575 Japan; 2 Department of Immunology, Tohoku University Graduate School of Medicine, 2-1 Seiryo, Aoba-ku, Sendai, Miyagi 980-8575 Japan; 3 Department of Cardiovascular Medicine, Tohoku University Graduate School of Medicine, 2-1 Seiryo, Aoba-ku, Sendai, Miyagi 980-8575 Japan; 4 Department of Pharmacology and Medicine and Center for Lung Biology, University of South Alabama, College of Medicine, 307 University Blvd N Mobile, AL 36688-0002 USA; 5 Department of Pathology, Ehime University Graduate School of Medicine, Shitsukawa, Toon, Ehime 791-0295 Japan; 6 Japan Science and Technology Agency, CREST, Tokyo, Japan; 7 Johnson & Johnson Pharmaceutical Research & Development, L.L.C., 3210 Merryfield Row, San Diego, California 92121, USA

## Abstract

**Background:**

Pulmonary hypertension (PH) refers to a spectrum of diseases with elevated pulmonary artery pressure. Pulmonary arterial hypertension (PAH) is a disease category that clinically presents with severe PH and that is histopathologically characterized by the occlusion of pulmonary arterioles, medial muscular hypertrophy, and/or intimal fibrosis. PAH occurs with a secondary as well as a primary onset. Secondary PAH is known to be complicated with immunological disorders. The aim of the present study is to histopathologically and genetically characterize a new animal model of PAH and clarify the role of OX40 ligand in the pathogenesis of PAH.

**Results:**

Spontaneous onset of PAH was stably identified in mice with immune abnormality because of overexpression of the tumor necrosis factor (TNF) family molecule OX40 ligand (OX40L). Histopathological and physical examinations revealed the onset of PAH-like disorders in the C57BL/6 (B6) strain of OX40L transgenic mice (B6.TgL). Comparative analysis performed using different strains of transgenic mice showed that this onset depends on the presence of OX40L in the B6 genetic background. Genetic analyses demonstrated a susceptibility locus of a B6 allele to this onset on chromosome 5. Immunological analyses revealed that the excessive OX40 signals in TgL mice attenuates expansion of regulatory T cells the B6 genetic background, suggesting an impact of the B6 genetic background on the differentiation of regulatory T cells.

**Conclusion:**

Present findings suggest a role for the OX40L-derived immune response and epistatic genetic effect in immune-mediated pathogenesis of PAH.

## Background

Pulmonary hypertension (PH) is a severe disease condition that can lead to progressive right ventricular failure and ultimately to death. Pulmonary arterial hypertension (PAH) is a major class of PH defined in the classification of the World Health Organization (WHO). The main histopathological manifestations of PAH are vasoconstriction, endothelial cell proliferation and fibrosis, smooth-muscle cell proliferation, and thrombosis in small pulmonary arteries. These changes result in elevation of pulmonary vascular resistance and, consequently, in pulmonary arterial pressure [1].

PAH occurs as either a primary (idiopathic or familial) or a secondary disease. According to the WHO classification, inflammatory conditions, such as collagen vascular diseases, and viral infections are associated with the occurrence of PAH. Indeed, patients with a subset of idiopathic PAH have some inflammatory disturbances, presented as elevated circulating levels of TNF-α, interleukin (IL)-1, and IL-6 [2]. In the case of severe PAH in humans, infiltration of immune cells, including T cells, B cells, and macrophages, is occasionally observed in pulmonary vascular lesions [3]. Most of the CD4^+ ^and CD8^+ ^T cells infiltrating into the intimal lesions have been shown to express effector memory T-cell markers, indicating the active status of the T cells. In animal models, augmented expression of IL-18 or administration of IL-6 is sufficient to induce mild spontaneous PH [4,5]. In the former case, IL-13 has been shown to critically mediate inflammatory signals in the lung. Recent studies have proposed that naturally arising CD4^+^CD25^+ ^regulatory T (T_reg_) cells, or their mediators, may inhibit the development of experimental PH [6]. Furthermore, it has been suggested that the deficiency of CD4^+ ^T cells in humans (e.g., in cases of HIV infection), or the depletion of CD4^+ ^T cells in experimental animal models, is associated with the development of PAH [7]. These observations implicate an immune-mediated mechanism in the development of PAH.

Signals through T-cell costimulatory molecules are critically involved in eliciting optimal T-cell functions [8]. OX40 (TNFRSF4, CD134) is a member of the TNF receptor superfamily that is transiently expressed on activated T cells. The ligand of OX40 (OX40L: TNFSF4, CD134L) is mainly expressed on mature antigen-presenting cells as well as on vascular endothelial cells [9-12]. The OX40-OX40L interaction is required for optimal effector function of T cells [13,14] and generation of memory T cells [15-18]. Recently, growing evidence has unveiled the importance of OX40 signals in the accumulation of effector CD4^+ ^T cells at inflammation sites in mouse models of autoimmune diseases. Moreover, a recent study has demonstrated that constitutive OX40-OX40L interactions in OX40L transgenic mice entail spontaneous development of ulcerative colitis-like disease and an undetermined lung disease, which is accompanied by significant production of an anti-DNA antibody [19]. Interestingly, these pathological manifestations have been observed in mice with the C57BL/6 (B6) genetic background but not in those with the BALB/c (BALB) genetic background. The strain-specific pathological manifestations implicate the presence of a genetic predisposition that modulates OX40L-dependent inflammation in the colon and lungs.

The goal of this study was to characterize the undetermined lung disease presented in an OX40L-transgenic B6 strain (B6.TgL) of mice. In the present study, we proposed a new spontaneous model for PAH. Furthermore, this study provided novel insight into the role of the OX40L-derived signal and the genetic predisposition in the immune-mediated pathogenic mechanism of PAH.

## Methods

### Mice

Mice with OX40L transgene under the expression control of *lck *promoter were generated in a C57BL/6 genetic background as described previously (B6.TgL) [19]. To generate OX40L transgenic mice on BALB/c background (BALB.TgL), B6.TgL backcrossed to BALB/c strains more than 8 times. Age and sex-matched wild-type C57BL/6 and BALB/c were used as controls. For genetic analyses, TgL mice with mixed genetic background were prepared by the mating of BALB × B6.TgL and (BALB × B6) F1 × B6.TgL. All mice were bred and maintained in conventional clean room in the animal department of the Oriental Bio-service, Co. Ltd, Shizuoka, Japan. In all animal experiments in this study, we followed the Tohoku University guidelines for animal experimentation.

### Histopathological examinations

At 20 weeks of age, each mouse was killed under ether anesthesia. The whole lung was immersion fixed in 10% formalin in 0.01 M phosphate buffer (pH 7.2), and embedded in paraffin. Tissue sections were stained with hematoxylin and eosin (H&E) and Masson's trichrome for light-microscopic examination. The disease score of PAH was histopathologically determined. Ten small pulmonary arteries along with terminal bronchioles were individually graded under microscopic examination according to following histopathological criteria: 0, normal; 1, significant, slight thickening of the media; 2, thickening of the media with intimal (endothelial) proliferation and/or fibrosis. A mean grade of all points examined was considered as an individual PAH score. Immunohistochemical analyses were performed using the primary antibodies to human α-smooth muscle actin (αSMA) (DACO, Tokyo, Japan), which has been shown to react mouse αSMA, and mouse CD31 (Santa Cruz Biotechnology, Santa Cruz, CA).

### Right ventricular systolic pressure measurements

B6, BALB, B6.TgL, and BALB.TgL mice were anesthetized by intraperitoneal injection of ketamine hydrochloride (60 mg/kg) and xylazine (8 mg/kg) or, in the second series of measurement using B6, and BALB.TgL mice, pentobarbital sodium (50 mg/kg). Right ventricular systolic pressure (RVSP) was measured in spontaneously breathing mice by direct puncture of the right ventricle with a 25-gauge needle connected to a pressure transducer [20]. In the second series with the pentobarbital anesthetization, it was measured in artificially ventilated mice with median thoracotomy.

### Evaluation of right ventricular hypertrophy

The hearts isolated from B6, BALB, B6.TgL, and BALB.TgL mice were fixed in formalin and dissected into right ventricle (RV), left ventricle (LV), and interventricular septum (IVS). The dissected ventricles were carefully washed in saline to remove blood clots and separately weighted. Right ventricular hypertrophy was evaluated by the weight ratio of RV/(LV+IVS).

### Antibodies and flow cytometric analysis

Anti-CD3-FITC, anti-CD4-allophycocyanin, anti-CD25-allophycocyanin, anti-CD44-phycoerythrin (PE), anti-CD62L-FITC, and anti-IL-17-PE were purchased from BD Biosciences (San Diego, CA). Anti-mouse Foxp3-PE (FJK-16S) was purchased from eBioscience (San Diego, CA). Anti-mouse CD3ε (clone 2C11) used for T cell stimulation and anti-mouse CD16/32 (clone 2.4G2) used for Fc receptor blocking were purified from hybridoma-cultured supernatants in our laboratory. Cells were incubated with antibodies for 30 min at 4°C and then washed to remove unbound antibodies. All the samples were analyzed with a FACSCalibur™ flow cytometer and the CellQuest™ program (BD Biosciences).

### Preparation of lymphocyte culture and cytokine measurements

Single-cell suspensions were prepared from spleen and lungs of an 8 to 10 week-old mouse, in which the lung disease of interest is not developed. Lymphocytes in the lung were obtained by digesting minced lung tissues with 150 U/ml collagenase (Maeda Co. Ltd., Tokyo, Japan) as described previously [21]. The number of effector/memory and regulatory T cells were calculated based on the percentage of each subpopulation that was CD44^high^CD62L^low ^and CD4^+^Foxp3^+^, respectively, and the total cell number in each organ. Total lymphocytes isolated from lung tissues that contained equal number of effector/memory CD4^+^T cells (normalized based on absolute number of effector/memory CD4 T cells) were stimulated with soluble anti-CD3ε (10 μg/ml) at 37°C for the indicated time. IL-13 levels were assayed in cultured supernatants using ELISA kit for IL-13 (R&D Systems, Minneapolis, MN), according to the manufacturer's recommendations. The production of IL-17 in lymphocytes was detected by intracellular staining with anti-IL-17-PE following incubation of lymphocytes for 4 h with 50 ng/ml PMA, 500 ng/ml Ionomycin (Sigma-Aldrich, St. Louis, MO) in the presence of 10 μg/ml brefeldin A (Invitrogen, Carlsbad, CA).

### Genetic mapping

Genotypes of BCN2.TgL mice were determined by polymerase chain reaction (PCR) using genomic DNA prepared from the tail tip. The genotyping PCR was performed using standard reagent and the following conditions: 94°C for 5 min, 35 cycle of 94°C for 30 sec, 58°C for 30 sec, 72°C for 30 sec, and final extension 72°C for 5 min with the 98 microsatellite markers (additional file 1), which represent amplified fragment-length polymorphism between BALB and B6 strains. This genotyping provided full coverage of the mouse autosomes with the marker spaced an average of 12.5 cM apart and a maximum distance of 35 cM between any two markers. PCR products were visualized with electrophoresis on 2-4% agarose gels containing 0.01% ethidium bromide.

In a genome-wide scan, we determined genotypes of the 48 BCN2.TgL mice, which were selected as the top (severest) 24 and the bottom 24 on the list of PAH score, at all the 98 microsatellite positions (additional file 1) . The association at each microsatellite position was evaluated with chi-square test for independence between the genotypes and the two groups that were positive and negative for the incidence of PAH, using standard 2 × 2 contingency matrices. A *p *value less than 0.05 was regarded as suggestive association. The suggestive association was confirmed by the two-tail *t*-test for the difference of means between the two genotype groups of a total of 341 BCN2.TgL mice. In this test a *P *value less than 0.0034 was regarded as suggestive association. This P threshold was referred to the previous recommendation [22].

In a linkage mapping, a linkage position was determined with the quantitative trait locus (QTL) program. The logarithm of odd (LOD) was determined with the interval mapping program in the Windows QTL Cartographer (V2.5) software. The PAH scores of all BCN2.TgL mice were used as an indicator of phenotype. A suggestively significant level (α = 0.05) of LOD was determined by the permutation test installed in this software (1000 permutations). Map positions (cM) of the microsatellite makers were based on the information of the Mouse Genome Database of The Jackson Laboratory (http://www.informatics.jax.org).

### Statistics

A 95% confidence interval shown in Table 1 was calculated using the method described previously [23]. The two-tailed *t*-test was used to evaluate a difference of means between two groups. A *P *value less than 0.05 considered as significant.

**Table 1:** Summary of PAH scores of B6, BALB, and transgenic strains of mice

Mice*		n†	Median of score	95% CI‡	Statistics§
B6		9	0	0 - 0.1	
BALB		2	0	n.d.	
B6. TgL		15	0.7	0.35 - 1.05	¶
BALB. TgL		8	0	0 - 0.1	
BCF1. TgL		30	0.05	0 - 0.1	
BCN2. TgL	female	174	0.25	0.2 - 0.4	¶
BCN2. TgL	male	167	0.25	0.2 - 0.4	¶

* All mice were killed at 20 week-old for this examination. BCF1, BALB × B6; BCN2, BCF1 × B6.TgL.

† Number of mice tested.

‡ CI, confidential interval. The CI was not determined (n.d.) for BALB/c (BALB).

§ The significance of this study was evaluated by Mann-Whitney U-test for the all strains.

¶, P < 0.01 (v.s. B6).

## Results

### Histopathological characterization of the lung phenotype

**Figure 1:**
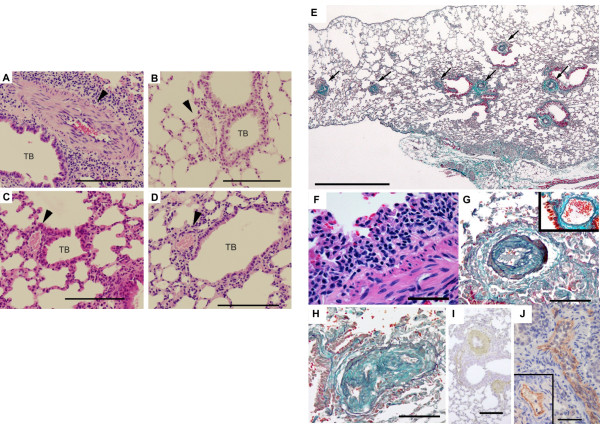
**Histopathological features of the lung disease in B6**.TgL mice. (A-D) Histopathological manifestation typically present in B6.TgL (A). No pathological manifestation observed in B6 (B), BALB.TgL (C), and BALB (D). The photograms indicated were taken from over 20-week aged male mouse. Arrow heads indicate pulmonary arteries. *TB*, terminal bronchiole. H&E staining. Scale bar = 100 μm. (E) Diffuse pathology present in B6.TgL. Masson's trichrome staining. Scale bar = 1 mm. (F) Perivascular lymphocytic infiltration in the affected lung. H&E staining. Scale bar = 50 μm. (G, H) Representative microscopic appearance in the affected arteries in the B6.TgL lung. An inset photogram in *G *represents the appearance of a normal pulmonary artery. Thickening of the intimal and, to a lesser extent, medial layers with marked intimal fibrosis is characteristic of the affected arteries. Masson's trichrome staining. Scale bar = 100 μm. *I *and *J*, expression of αSMA and CD31 (PECAM), respectively in the thickened arterial wall. The photogram in the inset of *J *represents a normal manifestation of unaffected artery. Immunohistochemical staining with hematoxylin counter-staining. Scale bar: in *I*, 200 μm; in *J*, 100 μm.

Microscopic examination revealed diffuse pathological changes in the small- to medium-sized pulmonary arteries in B6.TgL (Figure 1A, E), but not in B6 (Figure 1B), BALB strain of OX40L transgenic mice (BALB.TgL) (Figure 1C), or BALB (Figure 1D). This vascular lesion was found to be readily accompanied with perivascular infiltration of lymphocytes and, to a lesser extent, neutrophils (Figure 1F). These changes were mainly observed in arteries contained in bronchovascular bundles (respiratory arteries) and were typically characterized by fibrocellular endothelial proliferation of the intimal layer and, to a lesser extent, medial muscular hypertrophy (Figure 1G and 1H). Neither muscularization of distal pulmonary arterioles, which is a common pathological change in a hypoxic PAH model, nor plexiform lesions, found in human PAH, were identified. The cells in the intimal lesion were characterized as positive for a myofibroblast marker, smooth muscle-specific actin (αSMA) (Figure 1I), and an endothelial cell marker, CD31 (Figure 1J). When no treatment was administered to avoid vasospasm before the histopathological preparation, vasoconstriction was frequently observed in pulmonary arteries of B6.TgL but not in wild-type B6, BALB, and BALB.TgL mice, irrespective of the presence of the overt pathological changes mentioned above (data not shown). Perivascular lymphocytic infiltration was also observed around pulmonary veins; however, there was no pathologic remodeling in those veins as observed in the arteries (data not shown). Degenerative or granulomatous vascular lesions were not observed in conjunction with the perivascular infiltration, indicating that the vascular lesion of interest is not related to any type of vasculitis syndrome. We found no vascular lesions in the kidney or the colon of B6.TgL mice. Thus, the spontaneous lung disease in B6.TgL mice was characterized by lung-specific, pulmonary artery-restricted intimal thickening with lymphocytic (chronic) inflammation. These histopathological characteristics are similar yet distinct in a few points from those of human PAH.

### Strain-restricted onset of the lung disease

The PAH-like disease, as defined in the B6.TgL mice, was quantified with a PAH score in other strains of mice, including B6, BALB, BALB.TgL, BALB × B6.TgL (BCF1.TgL), and (BALB × B6) F1 × B6.TgL (BCN2.TgL). It was observed that wild-type and different TgL strains, such as BALB.TgL and BCF1.TgL, barely developed the PAH-like disease (Table 1). On the other hand, BCN2.TgL developed a PAH-like disease with a broader distribution of the PAH score than B6.TgL. There were no sex-related differences in the PAH scores (Table 1). These findings indicate that development of the PAH-like disease depends on both the effects of TgL and on an undefined B6-specific genetic background.

### Elevation of RV systolic pressure and RV hypertrophy in B6.TgL

**Figure 2:**
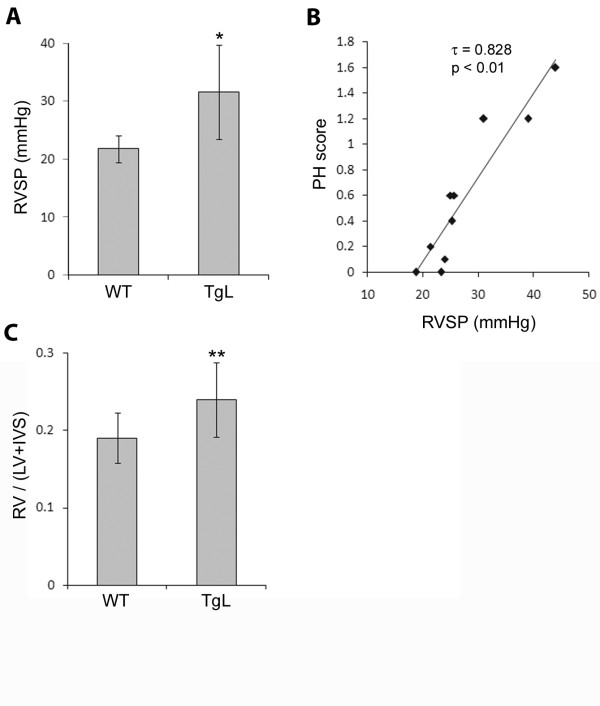
**Clinicopathological phenotypes associated with PH**. (A) Significant elevation of RVSP (mmHg) in B6.TgL. The result represents a mean ± standard deviation (SD) of 4 B6 or 6 B6.TgL mice (20 to 24 weeks old) for each group. (B) Correlation between RVSP values and PAH scores obtained from the 10 mice examined in *A*. The significance of this correlation was confirmed with the Kendall tau (τ) rank correlation coefficient (τ = 0.828). (C) RV hypertrophy manifested in B6.TgL. RV hypertrophy was evaluated with the index provided by the formula, RV/(LV+IVS). The result represents a mean ± SD of 12 B6 or 8 B6.TgL mice for each group. The significant difference between the two groups was evaluated by two-tailed *t*-test. *, p < 0.05; **, p < 0.01.

The PAH-like arteriopathy in B6.TgL mice indicated the onset of clinical PH. We therefore measured right ventricular (RV) systolic pressure (RVSP) in aged B6.TgL, BALB.TgL, and their wild-type strains. RVSP was significantly increased in the B6.TgL mice, as compared to B6 (Figure 2A). Importantly, the RVSP values were significantly correlated with the PAH scores (Figure 2B). Furthermore, significant RV hypertrophy was demonstrated for B6.TgL, as compared to B6 mice (Figure 2C). Increases in RVSP and RV hypertrophy were not observed in the BALB.TgL mice, as compared to B6 mice (additional file 2). In other experiments performed using wild-type BALB mice (30 w), there has been no evidence for RV hypertension or RV hypertrophy in BALB strain: RVSP = 21.3 ±2.72 mmHg, RV/(LV + IVS) = 0.25 ±0.026.

### Accumulation of effector/memory CD4^+ ^T cells in OX40L-Tg mice

**Figure 3:**
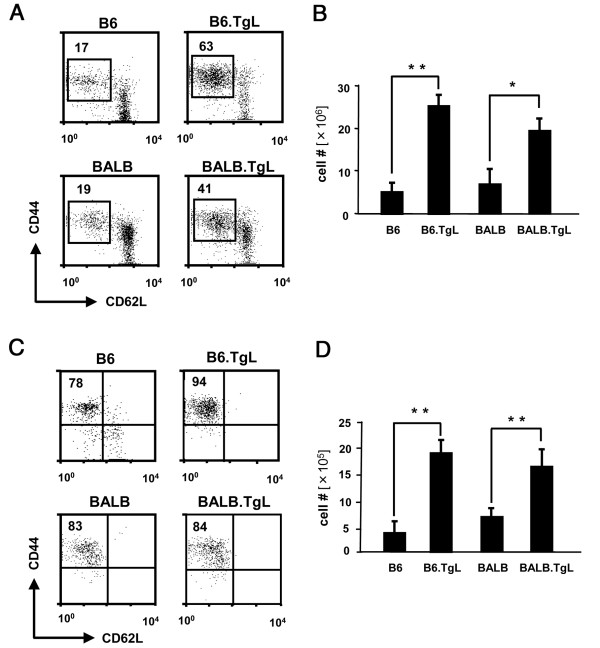
**Accumulation of effector/memory CD4^+ ^cells in transgenic (TgL) strains of mice**. The percentages of CD44^high^CD62L^low^CD25^-^CD4^+ ^T cells (effector/memory CD4^+^T cells) in a total of CD4^+^T cells are shown in the dot grams; (A) spleen, (C) lung. The absolute numbers of effector/memory CD4^+ ^T cells are shown in the bar grams; (B) spleen, (D) lung. The absolute number was calculated from the percentage of this subset and the total cell number in each organ. The results represent a mean ± SD of 6-8 mice per each group. The results of flow cytometry (A and C) are representative of three independent experiments performed using 8 to 10 week-old mice. The significant difference between the two groups was evaluated by two tailed *t-*test. *, *p *< 0.01; **, *p *< 0.001.

Previous studies performed with B6.TgL mice have demonstrated a selective increase in the number of CD44^high^CD62L^low ^effector/memory CD4^+ ^T cells in lymphoid and nonlymphoid tissues [17,19]. We examined whether the tissue distribution of effector/memory CD4^+ ^T cells was altered by the genetic background before the disease onset. Flow cytometric analyses revealed a significant increase in the number of effector/memory CD4^+ ^T cells in both the spleen (Figure 3A and 3B) and lungs (Figure 3C and 3D) in every TgL mouse examined. Importantly, this increase was not observed in a strain-specific manner, indicating that the development of PAH is not simply explained by the increase of effector/memory CD4^+ ^T cells.

### Strain-specific profile of cytokine production by the lung CD4^+ ^T cells

**Figure 4:**
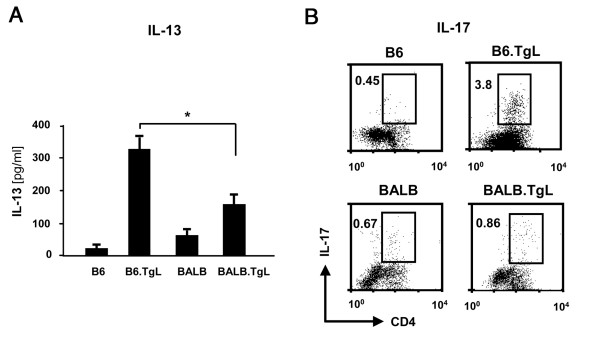
**Strain-specific cytokine profile in lung CD4^+^T cells**. (A) IL-13 production from a total of lymphocytes isolated from the lung of non-transgenic and transgenic (TgL) strains of mice at 8 to 10 week-old. Lymphocytes containing equal number of CD44^high^CD62L^low^CD4^+^T cells for each group (n = 4) of strain were stimulated with soluble anti-CD3 for 48 h and IL-13 in the cultured supernatant was quantified by ELISA. The significant difference between B6.TgL and BALB.TgL was evaluated by two-tailed *t*-test. *, p < 0.05. (B) IL-17 production in the lung CD4^+^T cells. Lymphocytes isolated as above were incubated with PMA/Ionomycin in the presence of brefeldin A for 4 h and intracellular IL-17 production was analyzed in CD4 T cells. Representative data from two independent experiments were shown.

The functionality of resident CD4^+ ^T cells in the lungs of TgL mice was determined by testing their ability to produce cytokines in response to anti-CD3 or PMA/ionomycin stimulation, respectively. Augmented IL-13 production was observed in the TgL-derived T cells and interestingly, this augmentation was greater in B6.TgL than in BALB.TgL (Figure 4A). Furthermore, a larger number of IL-17-producing CD4^+ ^T cells were observed in B6.TgL mice than in BALB.TgL (Figure 4B). IL-4 and IL-5 were not detected in stimulated lung cells by any strains (data not shown). These results indicate that the function of tissue resident CD4 T cells can be modulated by the excessive OX40 signals on B6 genetic background before disease onset.

### Over-expression of OX40L in B6 background alters the balance between lung resident effector/memory T cells and regulatory T cells

**Figure 5:**
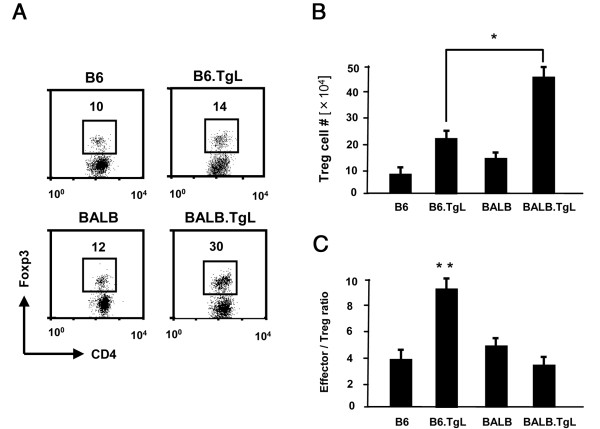
**Accumulation of T_reg _cells in non-transgenic and transgenic (TgL) strains of mice at 8 to 10 week-old**. The percentage (A) and absolute numbers (B) of CD4^+ ^Foxp3^+^T_reg _cells in the lung are shown. The absolute lymphocytic number was calculated from the percentage of T_reg _and the total cell number. (C) The ratio of effector/memory T cells (CD4+ CD25- CD44 ^high ^CD62L ^low^) to Fopx3+ T_reg _cells in the lung. A significant increase in this ratio is shown in B6.TgL strain of mice. The result represents a mean ± SD from 4 mice per each group. The results of flow cytometry (A) are representative of two independent experiments. The significant difference between the two groups was evaluated by two tailed *t-*test. *, *p *< 0.01; **, *p *< 0.001.

CD4^+^CD25^+^Foxp3^+ ^T cells, usually denoted as T_reg _cells, are known to control inflammatory responses by suppressing the activities of Foxp3^- ^effector T cells [24]. Several independent studies have demonstrated that lung resident T_reg _cells suppress type 2 immune responses and, consequently, reduce pulmonary inflammation [25-27]. We analyzed the population size of T_reg _cells, defined as CD4^+^Foxp3^+^, in the lung of non-transgenic and TgL strains. Flow cytometric analyses revealed that the frequency and absolute number of T_reg _cells increased in TgL strains in advance of the disease onset as compared with those in nontransgenic strains (Figure 5A and 5B). It was particularly noted that the increase of T_reg _was less in B6.TgL than in BALB.TgL, suggesting that B6-specific genetic factors counteract development of T_reg _cells in TgL mice. We also examined the ratio of Foxp3- effector/memory CD4 T cells (additional file 3) to Foxp3+ T_reg _before disease onset. The data shows an increased ratio of effector/memory T cells to T_reg _cells in the B6.TgL lung compared to the BALB.TgL lung (Figure 5C). These findings also suggest an important role of T_reg _in regulating inflammation associated with the pathogenesis of PAH-like disease in B6.TgL mice.

### Identification of a susceptibility locus for PAH

**Figure 6:**
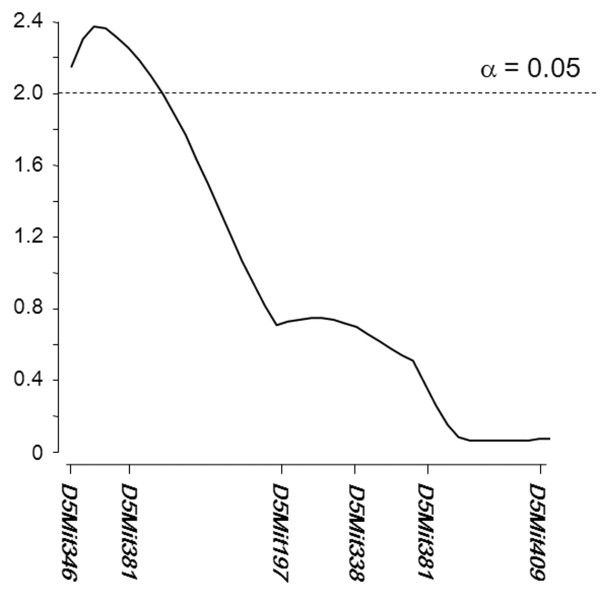
**LOD plots of the QTL analysis for PAH scores on chromosome 5**. The significant threshold level (α = 0.05) is shown by a score of 2 with a dashed line. The LOD peak (2.4) is located at 7 cM. The Y-axis denotes the level of the LOD score, and the X-axis denotes the genetic position defined by the microsatellite markers indicated: *D5Mit346 *(1 cM), *D5Mit381 *(8 cM), *D5Mit197 *(36 cM), *D5Mit338 *(59 cM), *D5Mit213 *(70 cM), and *D5Mit409 *(83 cM). The results were obtained using the interval mapping program of the MapMarker/QTL software.

TgL-dependent PH developed in a strain-specific manner, suggesting that the genetic background had an effect on the disease phenotypes. To identify a susceptibility locus for a PAH-like disease in B6.TgL, a genetic approach was employed using BCN2.TgL mice, which are descended from the B6.TgL and non-disease-prone BALB.TgL strains of mice. A genome-wide scan performed using selected 48 BCN2.TgL mice identified 4 candidate loci on chromosomes 5, 9, 13, and 17, which were possibly associated with the incidence of a PAH-like disease (additional file 1). The association study with 341 BCN2.TgL mice confirmed the suggestive association at *D5Mit346 *(1 cM) and *D5Mit381 *(8 cM) on chromosome 5 (Table 2). The other candidate loci preliminarily defined on chromosomes 9, 13, and 17 were not confirmed by this study. A QTL analysis consistently demonstrated a suggestive linkage between the level of PAH score and the chromosomal region between *D5Mit346 *and *D5Mit381*. This linkage was observed in a single LOD peak of 2.4 at 7 cM on chromosome 5 (Figure 6).

**Table 2:** Genetic association of PAH score in BCN2

Marker	Position (CM)	Mean of grade†	Mean of grade	P value‡
		BB	BC	
D5Mit346	1	0.51 ± 0.61 (172)	0.32 ± 0.46 (169)	0.0018 §
D5Mit381	8	0.51 ± 0.59 (178)	0.32 ± 0.46 (163)	0.0012 §
D5Mit197	36	0.46 ± 0.56 (192)	0.36 ± 0.52 (149)	0.0844
D5Mit338	59	0.45 ± 0.55 (191)	0.37 ± 0.53 (150)	0.1476
D5Mit213	70	0.41 ± 0.52 (192)	0.42 ± 0.58 (149)	0.9149
D5Mit409	83	0.42 ± 0.55 (165)	0.41 ± 0.54 (178)	0.766

* Values indicate mean ± SD and the values in parenthesis denote the number of mice.

† BB = B6 homozygote; BC = B6/BALB heterozygote

‡ The two-tail t-test. §, suggestive linkage [22].

## Discussion

Previous studies have shown that B6.TgL mice display abnormal T-cell differentiation and functions, and spontaneous inflammation in the colon and lung. The colonic phenotype in B6.TgL mice was histopathologically defined as an inflammatory bowel disease resembling ulcerative colitis in humans. In the present study, the undetermined lung disease in B6.TgL mice was characterized as a PAH-like disease. PAH is a clinical category of PH that comprises many different disease entities. Pathological manifestations of the PAH-like disease in B6.TgL mice are not completely parallel to those of idiopathic PAH. The differences between idiopathic PAH and the present animal model include the caliber of the affected arteries, the primarily affected layer of vascular wall, and the participation of massive lymphocytic perivascular infiltration. Further investigations are needed to define the present lung pathology as any type of PAH. An increasing body of evidence implicates the role of immune-mediated mechanisms in the pathogenesis of PAH. A type of PAH occurs secondarily to collagen vascular disorders, such as systemic sclerosis and mixed connective tissue disease (MCTD). Interestingly, PAH with MCTD presents with a prominent characteristic of endothelial degeneration and proliferation, probably due to the pathogenic contribution of autoantibodies to endothelial cells [28,29]. This characteristic may be a pathological consequence of immune-mediated mechanisms shared with the present model. The findings in the B6.TgL mice provide a possible insight into an implication of an OX40L-derived signal in the immune-mediated mechanism of endothelial pathology in PAH.

PAH is associated with endothelial cell dysfunction and vasoconstriction. There is no direct evidence for a link between these pulmonary vascular manifestations and abnormality *in situ *of OX40L-derived signal. However, it has been shown that OX40L-derived signals have a pathologic impact on the endothelial cell functions of systemic arteries. Recent studies have demonstrated an association of OX40L gene polymorphism with the susceptibility to atherosclerosis in humans [30], and the critical contribution of OX40-OX40L interactions to atherogenesis in low-density lipoprotein receptor-deficient mice [31]. The endothelial cells of the systemic arteries and those of the pulmonary arteries are exposed to different conditions, i.e., blood pressure and oxygen tension. It is interesting to know whether the OX40-OX40L interactions yield a different response on pulmonary endothelial cells than on systemic endothelial cells, and whether the OX40L gene polymorphism is associated with any type of PAH in humans.

Our present immunological studies performed using TgL and non-TgL strains of mice with different genetic backgrounds--B6 and BALB--revealed the respective effects of TgL and strain-dependent genetic background on immune phenotypes in the lung. Previous studies have demonstrated that OX40L-derived signals promote the expansion of effector/memory CD4^+ ^T cells [17,19] and naturally arising T_reg _cells [32], and enhance the production of IL-13 and IL-17 by CD4^+ ^T cells [33-35]. To examine which TgL-dependent immune aberrations are correlated with the onset of the PAH-like disease, we examined TgL-dependent immune phenotypes in the lungs of 2 different strains at a pre-disease stage. The findings indicate that B6-specific genetic factors influence the expansion of effector/memory CD4^+ ^T cells and T_reg _cells in advance of the onset of lung disease. A possible role of T_reg _cells has been documented in the development of PAH in humans [6]. Furthermore, it is clearly shown that B6-specific genetic factors increase the number of IL-17-producing CD4^+ ^T cells as well as secretion of IL-13 and IFNγ (data not shown) by lung tissue resident CD4^+ ^T cells. IL-17 producing CD4 T cells, namely Th17 cells are well known that participates in the pathogenesis of various organ-specific autoimmune diseases, such as inflammatory bowel disease and rheumatoid arthritis [36,37]. Although the role of Th17 cells in PAH in humans has not been determined, our present findings suggest that they indeed play a role in PAH. IL-13 serves as an important mediator in pulmonary inflammation [5,38,39], suggesting a causal contribution of IL-13 to the pathogenesis of the present model. The presence of immunological findings provides an insight into PAH-prone immune condition in the lung: the increase of proinflammatory effectors, IL-13 and Th17, and the decrease of an anti-inflammatory effector, T_reg_.

The present genome-wide genetic approach demonstrated a new susceptibility locus controlling the onset of a PAH-like disease in our model. Previous genetic studies performed on familial PAH have shown mutations in 2 genes responsible for susceptibility to PAH: bone morphogenetic protein receptor 2 gene (*BMPR2*) [40] and activin-like kinase type-1 gene (*ALK-1*) [41]. Our identified locus includes neither of these genes, nor, to the best of our knowledge, any gene involved in their signal transduction pathways. However, *Nos3 *and *Hgf *genes were particularly noted within this locus. Nitric oxide (NO) is known as a potent endothelial cell-derived vasodilator and an inhibitor of smooth muscle proliferation. Endothelial NO production largely depends on NOS3/eNOS (encoded by *Nos3*). NOS3-deficient mice showed reduced pulmonary vascular proliferation and remodeling to chronic hypoxia [42,43]. Several studies have reported the preventive role of NO in the development of PH in mice and humans. The polymorphism of human *Nos3 *gene is associated with high-altitude pulmonary edema and PH in patients with chronic obstructive pulmonary disease [44]. On the other hand, *Hgf*, which encodes hepatocyte growth factor (HGF), suppresses vascular medial hyperplasia and matrix accumulation in advanced PH in rats [45]. These findings have underscored the role of NOS3/eNOS or HGF as a pathogenic modifier in the present PH model.

A T-cell subset, type II helper T cell (Th2), plays an important role in the pathogenesis of PAH in mice [39]. In this regard, it is noteworthy that the 2 loci (the transgene locus and the susceptibility locus) have a strong impact on Th1/Th2 balance. The OX40 signal promotes a Th2-prone condition in mice [34]. On the other hand, NO and HGF serve as inducible factors for type I helper T cells (Th1) [46,47]. Therefore, in the TgL strains of mice, the 2 loci are mutually counterbalanced, and the net Th1/Th2 proportion depends mainly on the polymorphic effect of the susceptibility locus. In a B6 genetic background, an effect of the susceptibility locus may suppress Th1 responses and maximize Th2 augmentation in the lung conferred by the OX40L transgene, resulting in the B6-specific onset of PH.

## Conclusion

The present study reported a novel transgenic mouse model for PH. This model differs from previous PH models, which include a hypoxia-induced model, a drug-induced model, and a genetic model (i.e., endothelin B receptor-deficient) [48], in etiology, histopathology, and spontaneity of PH. Considering the physiological functions of OX40L, it is likely that the development of PH in the present model depends on Th2-mediated mechanisms. The present model may provide a new experimental opportunity for investigating immune-mediated mechanisms underlying PAH and the development of immune-targeted therapy for PAH.

## List of Abbreviations

αSMA: Alpha smooth muscle actin; B6**.**TgL: OX40L transgenic on C75BL/6 strain; BALB**. **TgL: OX40L transgenic on BALB/c strain; H & E: Hematoxylin and Eosin; HGF: Hepatocyte growth factor; IVS: Intraventricular Septum; LOD: Logarithm of odd; LV: Left ventricular; MCTD: Mixed connective tissue disease; NO: Nitric oxide; OX40L: OX40 ligand; PH: Pulmonary hypertension; PAH: Pulmonary arterial hypertension; PMA: phorbol 12-myristate 13-acetate; QTL: quantitative trait locus; RV: Right ventricle; RVSP: Right ventricular systolic pressure; Th: T helper cells; T reg: regulatory T cell; TNFSF: Tumor necrosis super-family

## Competing interests

The authors declare that they have no competing interests.

## Authors' contributions

MR and MO (Ono) conceived the project and contributed to all the aspect of this research. MN contributed to the genetic findings. MY, MO (Oka), and IFM contributed to histopathological findings. PS, NI, and KS (Sugamura) contributed to immunological findings. KS (Satoh) and HS contributed to physical findings such as blood pressure measurements. All authors read and approved the final manuscript.

## Supplementary Material

Additional file 1 **Summary of genome wide scan**. Genotypes of BCN2.TgL mice were determined by polymerase chain reaction (PCR) using genomic DNA for 98 microsatellite positions. Click here for file

Additional file 2 **Pathological phenotypes in the lung of BALB.TgL mice**. (A) RVSP (mmHg) in BALB.TgL (35 w, male, n = 4) and wild-type B6 (28 w, male, n = 4). The difference in the average values between the two strains is not statistically significant (p = 0.37, two tailed t test). These RVSP values tended to be lower than those in our previous measurement shown in Figure 2A. This change is probably due to the difference in the experimental conditions. (B) Evaluation of RV hypertrophy in BALB.TgL (35 w, male, n = 5) and wild-type B6 (28 w, male, n = 5). RV hypertrophy was evaluated with the index of RV/(LV+IVS). The difference between the two strains is not statistically significant (p = 0.76, two-tailed t-test). Click here for file

Additional file 3 **Foxp3 expression on total CD4 versus CD25 negative effector CD4 T cells**. Total CD4 and CD4+CD62L^low^CD25 negative cells from the lung tissue were stained for intracellular Foxp3. Click here for file
