# Supplementary material for: Indispensable roles of OX40L-derived signal and epistatic genetic effect in immune-mediated pathogenesis of spontaneous pulmonary hypertension
Source: BMC Immunol. 2011 Dec 15;12:67. doi: 10.1186/1471-2172-12-67 (PMC3269997; doi:10.1186/1471-2172-12-67)
Supplement: Additional file 1 — Summary of genome wide scan. Genotypes of BCN2.TgL mice were determined by polymerase chain reaction (PCR) using genomic DNA for 98 microsatellite positions. [file 1471-2172-12-67-S1.PDF]

Supplementary table 1. Summary of genome-wide scan [A]

Ch	Marker	cM	PAH$^+$		PAH$^-$		χ^2	p^C	
			BB[B]	*BC*	*BB*	*BC*			
1	*D1Mit231*	12	12	12	11	13	0.08	0.77	
	D1Mit302	32.8	13	11	9	15	1.34	0.25	
	D1Mit132	43.1	13	11	11	13	0.33	0.56	
	D1Mit471	63.6	13	11	11	13	0.33	0.56	
	D1Mit219	81.6	16	8	11	13	2.12	0.15	
	D1Mit113	93.3	15	9	13	11	0.34	0.56	
	D1Mit511	109.6	16	8	13	11	0.78	0.38	
2	*D2Mit1*	1	20	4	20	4	0.00	1.00	
	D2Mit365	17	15	9	9	15	3.00	0.08	
	D2Mit249	47.5	16	8	11	13	2.12	0.15	
	D2Mit423	68.9	16	8	11	13	2.12	0.15	
	D2Mit282	83	16	8	10	14	3.02	0.08	
	D2Mit229	99	15	9	15	9	0.00	1.00	
3	*D3Mit265*	4.6	13	11	10	14	0.75	0.39	
	D3Mit65	23.3	12	12	11	13	0.08	0.77	
	D3Mit28	43.6	11	13	9	15	0.34	0.56	
	D3Mit258	70.3	10	14	8	16	0.36	0.55	
	D3Mit19	87.6	13	11	8	16	2.12	0.15	
4	*D4Mit181*	2.5	16	8	16	8	0.00	1.00	
	D4Mit288	28.6	14	10	13	11	0.08	0.77	
	D4Mit152	40	14	10	12	12	0.34	0.56	
	D4Mit187	49.6	14	10	13	11	0.08	0.77	
	D4Mit251	66	11	13	10	14	0.08	0.77	
	D4Mit254	82	10	14	10	14	0.00	1.00	
5	*D5Mit346*	1	17	7	9	15	5.37	**0.02**	*
	D5Mit381	8	16	8	8	16	5.33	**0.02**	*
	D5Mit197	36	13	11	14	10	0.08	0.77	
	D5Mit338	59	12	12	13	11	0.08	0.77	
	D5Mit213	70	11	13	14	10	0.75	0.39	
	D5Mit409	83	11	13	13	11	0.33	0.56	
6	*D6Mit159*	7	10	14	13	11	0.75	0.39	
	D6Mit149	46.3	17	7	16	8	0.10	0.76	
	D6Mit339	65.5	14	10	15	9	0.09	0.77	

7	*D7Mit77*	9.4	12	12	16	8	1.37	0.24	
	D7Mit229	23	10	14	13	11	0.75	0.39	
	D7Mit301	46.5	12	12	13	11	0.08	0.77	
	D7Mit105	63.5	9	15	13	11	1.34	0.25	
	D7Mit46	69	10	14	13	11	0.75	0.39	
8	*D8Mit3*	8	10	14	14	10	1.33	0.25	
	D8Mit293	21	11	13	13	11	0.33	0.56	
	D8Mit178	33	11	13	12	12	0.08	0.77	
	D8Mit240	43	10	14	12	12	0.34	0.56	
	D8Mit200	58	14	10	13	11	0.08	0.77	
	D8Mit156	73	14	10	13	11	0.08	0.77	
9	*D9Mit247*	17	17	7	10	14	4.15	**0.04**	*
	D9Mit328	23	17	7	10	14	4.15	**0.04**	*
	D9Mit336	35	15	9	10	14	2.09	0.15	
	D9Mit355	53	13	11	11	13	0.33	0.56	
	D9Mit214	62	12	12	10	14	0.34	0.56	
	D9Mit18	71	12	12	11	13	0.08	0.77	
10	*D10Mit189*	7	12	12	13	11	0.08	0.77	
	D10Mit274	34	12	12	14	10	0.34	0.56	
	D10Mit162	59	12	12	13	11	0.08	0.77	
	D10Mit102	69	9	15	13	11	1.34	0.25	
11	*D11Mit227*	2	12	12	12	12	0.00	1.00	
	D11Mit367	20	14	10	13	11	0.08	0.77	
	D11Mit242	31	13	11	17	7	1.42	0.23	
	D11Mit327	52	10	14	15	9	2.09	0.15	
	D11Mit360	64	10	14	15	9	2.09	0.15	
	D11Mit338	75	10	14	15	9	2.09	0.15	
12	*D12Mit105*	6	8	16	12	12	1.37	0.24	
	D12Mit36	28	9	15	13	11	1.34	0.25	
	D12Mit14	37	8	16	12	12	1.37	0.24	
	D12Mit27	52	13	11	14	10	0.08	0.77	
	D12Mit292	58	12	12	11	13	0.08	0.77	
13	*D13Mit16*	10	15	9	11	13	1.34	0.25	
	D13Mit233	45	15	9	17	7	0.38	0.54	
	D13Mit291	61	13	11	18	6	2.28	0.13	

	D13Mit78	75	11	13	19	5	5.69	**0.02**	*
14	*D14Mit109*	3	12	12	17	7	2.18	0.14	
	D14Mit62	18.5	9	15	15	9	3.00	0.08	
	D14Mit113	25	9	15	12	12	0.76	0.38	
	D14Mit69	43	9	15	11	13	0.34	0.56	
	D14Mit266	60	13	11	11	13	0.33	0.56	
15	*D15Mit265*	12	17	7	13	11	1.42	0.23	
	D15Mit121	24	10	14	14	10	1.33	0.25	
	D15Mit189	48.5	8	16	13	11	2.12	0.15	
16	*D16Mit9*	4	10	14	10	14	0.00	1.00	
	D16Mit57	21.5	8	16	11	13	0.78	0.38	
	D16Mit76	43	8	16	11	13	0.78	0.38	
	D16Mit106	71.45	8	16	10	14	0.36	0.55	
17	*D17Mit115*	24.2	15	9	21	3	4.00	**0.05**	*
	D17Mit152	37.7	13	11	22	2	8.55	**0.003**	*
	D17Mit221	56.7	14	10	17	7	0.82	0.37	
18	*D18Mit197*	6	10	14	13	11	0.75	0.39	
	D18Mit9	42	12	12	13	11	0.08	0.77	
	D18Mit210	47	10	14	15	9	2.09	0.15	
19	*D19Mit68*	6	13	11	12	12	0.08	0.77	
	D19Mit41	16	14	10	14	10	0.00	1.00	
	D19Mit46	24	9	15	11	13	0.34	0.56	
	D19Mit71	54	11	13	12	12	0.08	0.77	

A Values represent the number of mice. PAH^+, mice with high PAH score; PAH^-, mice without PAH.

B *BB* = B6 homozygote; *BC* = B6/BALB heterozygote.

C Two-tail *t*-test. A *P* value < 0.05 (*) was regarded as candidate association.
