# Supplementary figures and images for: Indispensable roles of OX40L-derived signal and epistatic genetic effect in immune-mediated pathogenesis of spontaneous pulmonary hypertension
Source: BMC Immunol. 2011 Dec 15;12:67. doi: 10.1186/1471-2172-12-67 (PMC3269997; doi:10.1186/1471-2172-12-67)

**A**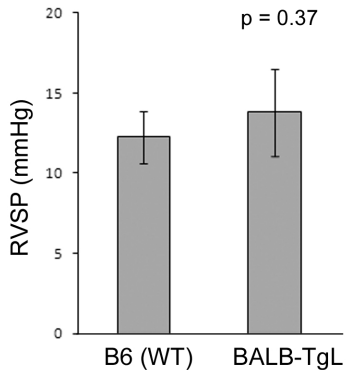**B**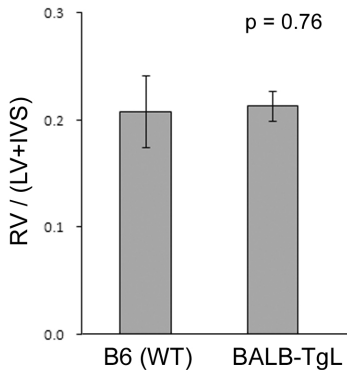

Supplement: Additional file 2 — Pathological phenotypes in the lung of BALB.TgL mice. (A) RVSP (mmHg) in BALB.TgL (35 w, male, n = 4) and wild-type B6 (28 w, male, n = 4). The difference in the average values between the two strains is not statistically significant (p = 0.37, two tailed t test). These RVSP values tended to be lower than those in our previous measurement shown in Figure 2A. This change is probably due to the difference in the experimental conditions. (B) Evaluation of RV hypertrophy in BALB.TgL (35 w, male, n = 5) and wild-type B6 (28 w, male, n = 5). RV hypertrophy was evaluated with the index of RV/(LV+IVS). The difference between the two strains is not statistically significant (p = 0.76, two-tailed t-test). [file 1471-2172-12-67-S2.PDF]

Total CD4<sup>+</sup>

CD4<sup>+</sup> CD62L<sup>low</sup> CD25<sup>-</sup>

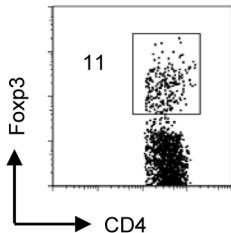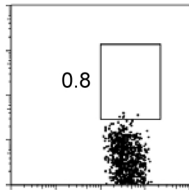

Supplement: Additional file 3 — Foxp3 expression on total CD4 versus CD25 negative effector CD4 T cells. Total CD4 and CD4+CD62LlowCD25 negative cells from the lung tissue were stained for intracellular Foxp3. [file 1471-2172-12-67-S3.PDF]
